# Positional Information‐Based Organization of Surfactant Droplet Swarms Emerging from Competition Between Local and Global Marangoni Effects

**DOI:** 10.1002/smll.202403720

**Published:** 2024-08-21

**Authors:** Pieter J. de Visser, Mink Neeleman, Pim F. J. Dankloff, Max T. G. M. Derks, Peter A. Korevaar

**Affiliations:** ^1^ Institute for Molecules and Materials Radboud University Heyendaalseweg 135 Nijmegen 6525 AJ The Netherlands

**Keywords:** adaptive behavior, amphiphiles, chemical gradients, marangoni effect, self‐organization

## Abstract

Positional information is key for particles to adapt their behavior based on their position in external concentration gradients, and thereby self‐organize into complex patterns. Here, position‐dependent behavior of floating surfactant droplets that self‐organize in a pH gradient is demonstrated, using the Marangoni effect to translate gradients of surface‐active molecules into motion. First, fields of surfactant microliter‐droplets are generated, in which droplets floating on water drive local, outbound Marangoni flows upon dissolution of surfactant and concomitantly grow myelin filaments. Next, a competing surfactant based on a hydrolysable amide is introduced, which is more surface active than the myelin surfactant and thereby inhibits the local Marangoni flows and myelin growth from the droplets. Upon introducing a pH gradient, the amide surfactant hydrolyses in the acidic region, so that the local Marangoni flows and myelin growth are reestablished. The resulting combination of local and global surface tension gradients produces a region of myelin‐growing droplets and a region where myelin growth is suppressed, separated by a wave front of closely packed droplets, of which the position can be controlled by the pH gradient. Thereby, it is shown how “French flag”‐patterns, in synthetic settings typically emerging from reaction‐diffusion systems, can also be established via surfactant droplet systems.

## Graphical abstract

Chemical gradients establish positional information to direct self‐organization of droplet‐based systems. Surfactant droplets are presented that grow myelin filaments and self‐organize in a pH gradient. A competing pH‐sensitive surfactant leads to position‐dependent myelin growth and droplet‐droplet repulsion, because of the emergence of two Marangoni flows: global chemo‐attraction and local repulsion. As a result, the surfactant droplets organize in “French flag”‐patterns.

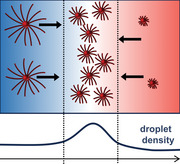

## 1 Introduction

Designing strategies that transform molecular systems into organization of matter at the mesoscale allows for autonomous behavior emerging from the bottom‐up.^[^ ^1^, ^2^ ^]^ Drawing inspiration from the behavior of living organisms, such as the formation of optimized networks in slime molds to efficiently distribute nutrients,^[^ ^3^, ^4^ ^]^ or the collective migration of social amoebae to survive in hostile environments,^[^ ^5^, ^6^, ^7^ ^]^ or the spontaneous formation of cell‐like compartments from cytoplasmic components,^[^ ^8^ ^]^ new classes of adaptive materials can be designed with the potential to function autonomously.^[^ ^9^, ^10^, ^11^ ^]^ At the same time, de novo‐designed material systems based on a minimalistic set of molecular building blocks allow us to unravel what physicochemical principles and feedback mechanisms are required for self‐organization. One essential principle is the presence of spatiotemporal chemical gradients, leading to persistent anisotropy in the system.^[^ ^12^, ^13^ ^]^ These gradients must be sustained actively to avoid thermodynamic equilibrium, i.e., to avoid a collapse into an isotropic, unorganized phase. Moreover, to translate chemical gradients into self‐organization at the meso‐ or macroscale, (supra)molecular building blocks responsive to these chemical gradients are required.

The Marangoni effect provides an effective mechanism to transform concentration gradients into motion of meso‐ and macroscale objects, driving the organization of such objects. Starting off with concentration gradients of surface active molecules at air–liquid interfaces, the Marangoni effect leads to interfacial flow from low toward high surface tension regions.^[^ ^14^, ^15^ ^]^ As a result, passively floating objects follow the Marangoni flow,^[^ ^16^ ^]^ such that global surface tension gradients result in accumulation of these objects in the high surface tension region. Introducing particles or droplets that release amphiphilic molecules extends the complexity of the self‐organization by generating local concentration gradients around these floating particles or droplets. The resulting local Marangoni flows result into mutual repulsion amongst the particles, driving their organization into spread‐out patterns.^[^ ^17^ ^]^ When combined with elasto‐capillary attractive effects, dynamic droplet clusters can be produced, that are typically both motile and continuously assemble and disassemble.^[^ ^18^, ^19^, ^20^ ^]^

A remaining challenge is to control the positioning of self‐organizing patterns based on Marangoni flows, as an alternative to either i) accumulation of all floating droplets at one location via global Marangoni flow, or ii) mutually repulsive spreading of droplets in a homogeneous field, via local Marangoni flows. This notion prompted us to establish a system of droplets in a global surface tension gradient that display different behavior (e.g., growth, motion) dependent on the local surface tension, and thereby generate differently populated regions along such gradients. In biological systems, Wolpert's concept of positional information processing describes how morphogen concentration gradients drive cellular differentiation into patterned tissues.^[^ ^21^, ^22^ ^]^ In synthetic systems, “French flag”‐patterns have been reported to emerge from chemical reaction networks that generate different product concentration levels dependent on the local concentration of a spatially varying reagent.^[^ ^23^, ^24^ ^]^ However, to the best of our knowledge, the spontaneous generation of such patterns based on surfactant systems and floating droplets has not been reported.

**Figure 1:**
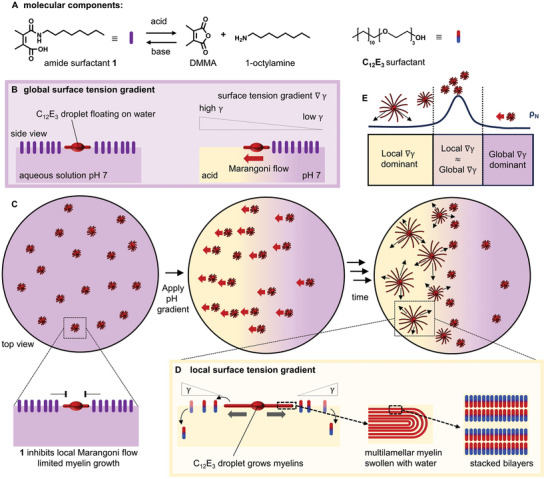
Self‐organization of floating droplets driven by the combination of global and local Marangoni flows. A) In our system, two surfactants are used, amide surfactant **1** that is formed from 2,3‐dimethylmaleic anhydride (DMMA) and 1‐octylamine, and tri(ethylene glycol) dodecyl ether (C_12_E_3_). B) **1** hydrolyses under acidic conditions, such that a pH gradient in an aqueous solution of **1** produces a global surface tension gradient, leading to a Marangoni flow towards the region of low amide surfactant **1** concentration (low pH). C) When C_12_E_3_ microdroplets are deposited at a solution of **1**, the strong surfactant **1** inhibits the local Marangoni flow that is typically generated around C_12_E_3_ droplets. D) In the acidic region, where **1** is hydrolyzed, C_12_E_3_ droplets start to generate local Marangoni flows (outbound from the droplets) that drive the growth of myelin filaments from the droplets, comprising stacked bilayers of C_12_E_3_ that are swollen with water. In the acidic region, these global Marangoni flows push the C_12_E_3_ droplets apart. E) As a result, a “French flag”‐pattern appears of subsequent low‐high‐low droplet density ρ_N_, determined by the balance between global and local surface tension gradients.

Here, we present floating surfactant droplets that produce “French flag”‐patterns when exposed to a pH gradient (**Figure** **1**). As the molecular components we use a pH‐sensitive surfactant **1** and tri(ethylene glycol) dodecyl ether (**C_12_E_3_**, *vide infra*). First, when exposed to a pH gradient, **1** is hydrolyzed in the acidic region, producing a global surface tension gradient that drives a global Marangoni flow towards the acidic side of the pH gradient. When liquid microliter‐droplets of C_12_E_3_ are deposited at the air–water interface, the global Marangoni flow will transfer these floating droplets towards the acidic side as well. Next, to establish local Marangoni flow, we exploit the surface‐active behavior of C_12_E_3_. Earlier, we have shown how floating C_12_E_3_ droplets release C_12_E_3_ to the air–water interface and generate persistent local Marangoni flows. Concomitant to these Marangoni flows, outbound from the droplets, C_12_E_3_ assembles into filaments, known as myelins, that have a thickness of ≈20–50 µm and are extruded from the droplets into millimeter‐long structures.^[^ ^25^, ^26^ ^]^ As **1** is more surface‐active than the myelin‐producing surfactant C_12_E_3_, it inhibits the local Marangoni flow and thereby suppresses the myelin formation. Hence, only upon hydrolysis of **1** in the acidic region, the local Marangoni flow and corresponding myelin growth can be reactivated.

By combining these effects, we show that for large swarms of C_12_E_3_ droplets under a pH gradient, the droplets’ positioning as well as their myelin growth is governed by the interplay of the global and local Marangoni flows. Initially, the hydrolysis of the pH‐sensitive surfactant generates a global Marangoni flow towards the acidic side of the pH gradient. Subsequently, the C_12_E_3_ droplets in the acidic region start to grow myelins and generate repulsive Marangoni flows – pushing neighboring droplets away from the acidic region. Together, the competition between these local repulsive interactions and the global, attractive Marangoni flow leads to the emergence of a self‐organized “French flag”‐pattern along the pH gradient. Going from low to high pH, we observe subsequent regions of sparse – dense – sparse droplet populations, as well as a decrease of the myelin corona size. Finally, we demonstrate that the sizes of these regions are determined by the amount of acid added to the solution.

## 2 Results and Discussion

### 2.1 Interfacial Competition Between C_12_E_3_ and Amide Surfactant 1

As amphiphile building block for the floating droplets, we selected the nonionic amphiphile tri(ethylene glycol) monododecyl ether (C_12_E_3_). Earlier, we reported that when deposited at an air–water interface, droplets of C_12_E_3_ absorb water and concomitantly form a “corona” of radially spreading filaments known as myelins,^[^ ^27^, ^28^ ^]^ as shown in Figure 1D. These myelins are extruded from the droplet by the outbound Marangoni flow, which is driven by slow release of C_12_E_3_ from the droplet to the air–water interface. We determined the associated surface tension for an aqueous C_12_E_3_ solution (500 µm, ≈10x cmc)^[^ ^29^ ^]^ at 27.9 ± 0.1 mN m^−1^. Hence, to inhibit the Marangoni flow generated by the release of C_12_E_3_, the surface tension of the competing dynamic surfactant adsorbed at the air–water interface should be ≤ 27.9 mN m^−1^.

**Figure 2:**
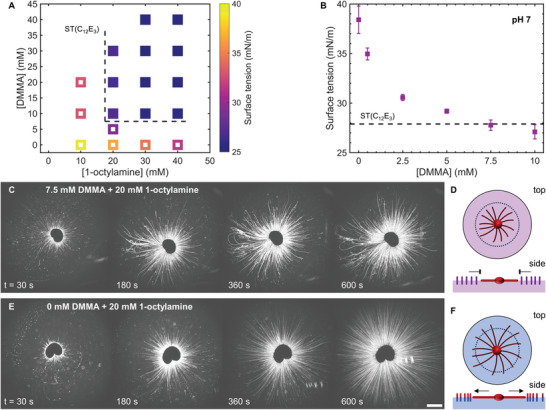
Amide surfactant **1** precursor concentrations required to inhibit C_12_E_3_ myelin growth. A) Surface tension at varying concentrations of DMMA and 1‐octylamine (*n* = 2). The dashed line indicates the approximate surface tension of C_12_E_3_, separating surface tension values higher than that of C_12_E_3_ (open symbols) and surface tension values lower than that of C_12_E_3_ (closed symbols). B) Average surface tension of amide surfactant **1** at a fixed concentration of 1‐octylamine (20 mm) and varying DMMA concentrations. The dashed line indicates the surface tension of C_12_E_3_. Error bars display the standard deviation (*n* = 3). C) Optical microscopy recordings of 1 µL C_12_E_3_ deposited on a solution with 20 mm 1‐octylamine and 7.5 mm DMMA, showing the inhibition of the myelin growth and the formation of a limited myelin corona that barely spans the field of view of the microscope, indicated by the dashed circle in scheme D. E) With a solution of 20 mm 1‐octylamine and no DMMA, such that no amide surfactant **1** could be formed, the myelins grow unhampered and the myelins grow longer than the field of view of the microscope, see scheme F. The scale bar indicates 2 mm. For all solutions, the pH was set to 7 with a 100 mm sodium phosphate buffer.

The competing amide surfactant **1** is created in situ in an aqueous solution from 1‐octylamine and 2,3‐dimethylmaleic anhydride (DMMA), as reported by Liu et al. (Figure 1A).^[^ ^30^ ^]^ We assessed the potential of amide surfactant **1** to inhibit myelin growth by measuring the surface tension of solutions with varying concentrations of the precursors 1‐octylamine and DMMA in a sodium phosphate buffer (PB, 100 mm) at pH 7. As shown in **Figure** **2A**, 1‐octylamine is surface active, although insufficiently to decrease the surface tension below 27.9 mN m^−1^, i.e., to compete with C_12_E_3_. Upon increasing the concentration of DMMA added to 1‐octylamine solutions, the surface tensions were observed to decrease. As DMMA is not surface active (Figure S1, Supporting Information), we ascribe this decrease to the surface activity of **1** that is formed. Gratifyingly, the formation of **1** is confirmed by ^1^H‐NMR (Figure S2, Supporting Information). Furthermore, at 1‐octylamine concentrations ≥ 20 mm and DMMA concentrations ≥ 10 mm, surface tensions below the value of C_12_E_3_ were obtained. As shown in Figure 2B, the surface tension of 1‐octylamine (20 mm) is ≈37 mN m^−1^, and decreases upon increasing the DMMA concentration to the point where it matches the surface tension of C_12_E_3_, at 7.5 mm.

Guided by the surface tension measurements, we assessed the capacity of **1** to inhibit the local Marangoni flow generated by a C_12_E_3_ droplet at the precursor composition of 20 mm 1‐octylamine and 7.5 mm DMMA (in 100 mm PB, pH 7). As shown in Figure 2C and Movie S1 (Supporting Information), the myelins initially grow from the C_12_E_3_ droplet (1 µL), but minutes after deposition of the droplet their growth comes to a halt, so that the myelin corona size stays constant. We note that both the time point at which the myelin growth halts as well as the final corona size varies from droplet to droplet (*vide infra*). Importantly, the myelin growth from a droplet deposited on a 20 mm 1‐octylamine solution, in the absence of DMMA, was observed to proceed monotonously (Figure 2E; Movie S1, Supporting Information). After ≈6 min, the corona fills the entire field of view of the microscope, corresponding to a corona diameter of ≈14.4 mm (note that the filaments do not grow that far in the presence of 7.5 mm DMMA, Figure 2C). Additionally, for C_12_E_3_ droplets deposited on a 7.5 mm DMMA solution, the myelin growth does not halt, although DMMA appears to affect the myelin density in the corona (Figure S3, Movie S1, Supporting Information). Together, our results show that the combination of 20 mm 1‐octylamine and 7.5 mm DMMA, at pH 7, produces sufficient amide surfactant **1** to outcompete C_12_E_3_ at the air–water interface, as indicated by the inhibited growth of myelins from the C_12_E_3_ droplet.

### 2.2 Amide Surfactant 1 Hydrolysis upon Addition of Acid

**Figure 3:**
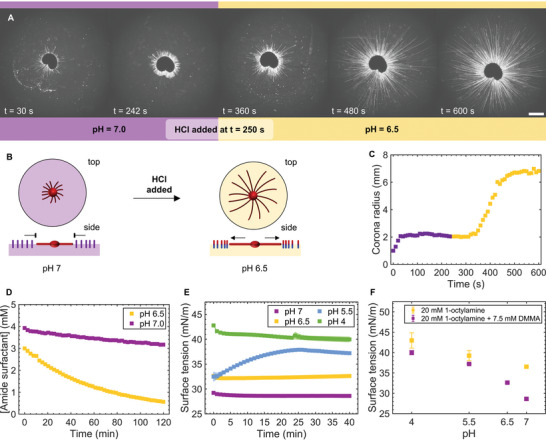
Amide surfactant **1** hydrolyses upon addition of acid. A) Optical microscopy recordings of a 1 µL C_12_E_3_ droplet deposited on a solution with 20 mm 1‐octylamine, 7.5 mm DMMA at pH 7 (100 mm PB). At t = 250 s, 85 µL 2 m HCl is added to lower the pH to 6.5 and trigger myelin growth. The scale bar indicates 2 mm. B) Scheme of filament growth upon pH change. C) Time‐dependent filament corona radius corresponding to the experiment in a. D) Time‐dependent concentration of amide surfactant **1**, measured by quantitative ^1^H‐NMR on a solution of 20 mm 1‐octylamine and 5 mm DMMA at pH 6.5 and 7.0 (90:10 100 mm PB:D_2_O). At the start of the measurement at pH 6.5, 30% of amide surfactant **1** has been hydrolyzed compared to the measurement at pH 7.0. E) Time‐dependent surface tension, measured on solutions of 20 mm 1‐octylamine and 7.5 mm DMMA at pH 7, 6.5, 5.5 and 4 (100 mm PB). The jump in surface tension at t = 0 upon decreasing the pH from 7 to 6.5 or 5.5, indicates that **1** hydrolyses rapidly upon addition of a concentrated acid solution, and after the solution has been mixed, it steadily hydrolyses slower depending on the pH. Error bars indicate standard deviation (*n =* 2). F) pH‐dependent surface tension of 20 mm 1‐octylamine and 7.5 mm DMMA compared to 20 mm 1‐octylamine, both at pH 7 (100 mm PB). Purple data points correspond to the final data points displayed in E. At pH 4 and 5.5, the surface tension is the same for either solution, indicating that no amide surfactant **1** is formed. Error bars indicate standard deviation (*n =* 2).

Next, we explored the dynamic covalent character of amide surfactant **1** to control the local Marangoni flow generated by C_12_E_3_ droplets. The amide surfactant is dynamic covalent due to the proximity of the carboxylic acid to the amide bond (Figure 1A), which makes the amide bond hydrolysable under weakly acidic conditions.^[^ ^30^, ^31^ ^]^ Hence, we hypothesized that addition of a small amount of acid hydrolyses the amide surfactant and thereby cancels the inhibition of the myelin growth. Starting off with a 1 µL C_12_E_3_ droplet deposited on an aqueous solution of 20 mm 1‐octylamine and 7.5 mm DMMA at pH 7 (100 mm PB), we observe inhibited myelin growth indicating that the local Marangoni flow is suppressed due to the presence of amide surfactant **1** (**Figure** **3A**). Next, addition of HCl (85 µL, 2 m) results in a rapid increase of the myelin growth after a lag phase of ≈2 min, resulting in a myelin corona with a radius comparable to experiments in which no amide surfactant **1** was present (Figure 2E). The same lag phase and subsequent increase in filament length upon addition of acid is clearly visible after applying image analysis on the microscopy images (Figure 3C). Before addition of acid, the corona radius including droplet is ≈2 mm. Upon addition of acid, it takes ≈100 s for the filament growth to pick up, after which the corona rapidly grows to fill the field of view of the camera ≈250 s after addition of acid. We measured the pH of the solution after addition of acid to be 6.5.

To assess the hydrolysis dynamics of amide surfactant **1**, we conducted both quantitative ^1^H‐NMR and time‐dependent surface tension measurements. At pH 7.0, we observe a very slow hydrolysis of amide surfactant **1** in ^1^H‐NMR (Figure 3D), whereas the surface tension stayed constant (7.5 mm DMMA: Figure 3E and 5 mm DMMA: Figure S4, Supporting Information). Upon addition of HCl (2 m) to a solution of DMMA and 1‐octylamine in phosphate buffer, to decrease its pH from 7.0 to 6.5, we observe a slow hydrolysis of **1** in ^1^H‐NMR. We observe that at pH 6.5, the surface tension is already higher at the start of the experiment compared to the surface tension at pH 7.0, and subsequently gives a very slow rise over time. The slow hydrolysis observed at pH 6.5 is in surprising contrast to the fast response in the start of the myelin growth upon the addition of acid displayed in Figure 3A, which led to a pH of 6.5 as well. We hypothesize that the rapid start of myelin growth is caused by imperfect mixing upon the addition of 2 m HCl, resulting in sections of the solution that temporarily have a pH that is much lower than pH 6.5, and thereby facilitate rapid hydrolysis of **1** at low pH – prior to the solution reaching a fully mixed state. For the ^1^H‐NMR experiment conducted on the solution of DMMA and 1‐octylamine at pH 6.5, we observed a similar phenomenon: compared to the concentration of **1** at pH 7.0, the concentration of **1** already declined by 30% at the start of the ^1^H‐NMR measurements, ≈1 min after addition of 2 m HCl and mixing to end up with a solution at pH 6.5. Gratifyingly, for solutions of **1** at pH 5.5, we observe a steady increase in surface tension in the first 25 min of measuring the surface tension. Moreover, for solutions of **1** at pH 4, we observed that right after their preparation – i.e. decreasing the pH from 7.0 upon the addition of 2 m HCl – the surface tension approximates the surface tension of 20 mm 1‐octylamine at this pH value, meaning that all amide surfactant **1** has rapidly hydrolyzed at low pH (Figure 3F).

Together, our results show that we can cancel the inhibitory effect of amide surfactant **1** on the local Marangoni flow and the corresponding myelin growth from C_12_E_3_ droplets, by addition of a small amount of concentrated acid to the system. To elicit this response, localized strong decrease of the pH is required to rapidly hydrolyze amide surfactant **1**.

### 2.3 Multi‐Droplet Deposition using an Automated Droplet Dispenser

**Figure 4:**
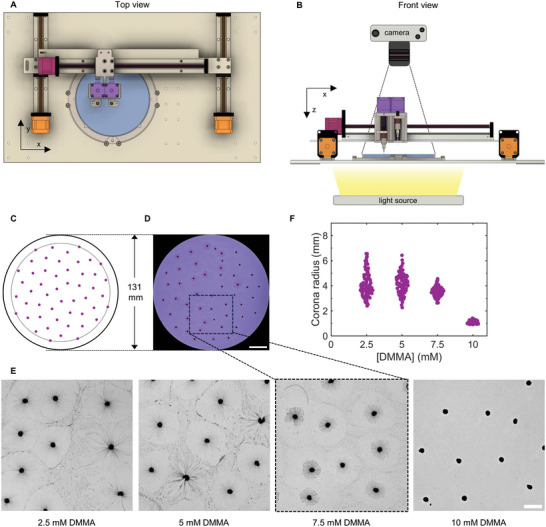
C_12_E_3_ droplet deposition with a dispensing robot. A,B) Drawing of the robot setup as seen from the top (A) and front (B). The robot controls the movement of two dispensing needles via motorized axes. Two parallel motorized axes (orange) actuate the needles in the y‐direction, on top of which a single motorized axis (red) is mounted that actuates the needles in the x‐direction. Both dispensing needles are individually actuated in the z‐direction by two independent motorized axes (purple). C) Droplet distribution of 50 droplets in the sunflower seed arrangement. The dashed circle indicates the maximum radial position of any droplet *r_max_* = 56.2 mm that ensures that droplets are not deposited too close to the dish edge. D) Photograph of 50 1 µL C_12_E_3_ droplets immediately after deposition on top of a solution of 20 mm 1‐octylamine, 7.5 mm DMMA, 0.0025 wt.% bromocresol purple at pH 7 (100 mm PB). The scale bar indicates 20 mm. E) Zoomed sections of photographs of 50 deposited droplets deposited on solutions with varying concentrations of DMMA that exemplify variation in the size of the myelin corona, see Figure S5 (Supporting Information). To enhance visibility of the filaments, the blue channel of the original RGB images was contrast enhanced. The scale bar indicates 10 mm. F) Filament corona sizes as a function of DMMA concentration of C_12_E_3_ droplets 5 min after deposition of 50 droplets on solutions with 20 mm 1‐octylamine at pH 7 (100 mm PB). Experiments were performed in duplo. The width of the data clouds indicates the approximate density of data points.

To study the self‐organization of droplets, as conceptualized in Figure 1, we developed a droplet dispensing robot that allows for rapid and reproducible deposition of a large number of C_12_E_3_ droplets at the air–water interface. The robot consists of motorized axes mounted to an aluminum base plate to translate a dispensing needle in 3 dimensions (x,y,z) (**Figure** **4**A,B). To dispense C_12_E_3_ droplets, another motorized axis is employed as a syringe pump, connected to the dispensing needle via PTFE tubing. The actions of all motorized axes and syringe pumps can be programmed via a controlling unit that is connected to a computer. The aqueous solution is contained in a circular glass dish (diameter = 131 mm) and supported on top of a circular opening in the base plate. The sample is illuminated by a light source from the bottom, and images of the sample are captured by a camera positioned above the sample (Figure 4B). To deposit C_12_E_3_ droplets precisely at the air–water interface, we use an electrical probing circuit, as described in the Methods section.

We deposit 1 µL C_12_E_3_ droplets on a solution of 20 mm 1‐octylamine and 7.5 mm DMMA at pH 7 (PB) in the glass dish. To accommodate each droplet with enough space to grow a myelin corona of ≈1.5–2 mm in diameter, comparable to previously observed myelin corona sizes (Figures 2C and 3A), the area of the glass dish (diameter = 131 mm) provides space for 50 droplets. To ensure that all droplets are homogeneously distributed, we deposit the 50 droplets in the sunflower seed arrangement, in which the angle between neighboring droplets is governed by the golden ratio φ = (1 +√5)/2 (Figure 4C). Even though the first few droplets move away from their position, the droplet distribution that is obtained after the deposition process, which takes ≈5 min, is homogeneous and comparable to the sunflower seed arrangement (Figure 4D). The myelin coronas of the deposited droplets vary in size (Figure 4E), with relatively small corona size variations for solutions with 7.5 or 10 mm DMMA compared to solutions with 2.5 or 5 mm DMMA (Figure 4F; Figure S5, Supporting Information).

### 2.4 Spatial Differentiation of Droplet Swarms based on Positional Information in a pH Gradient

**Figure 5:**
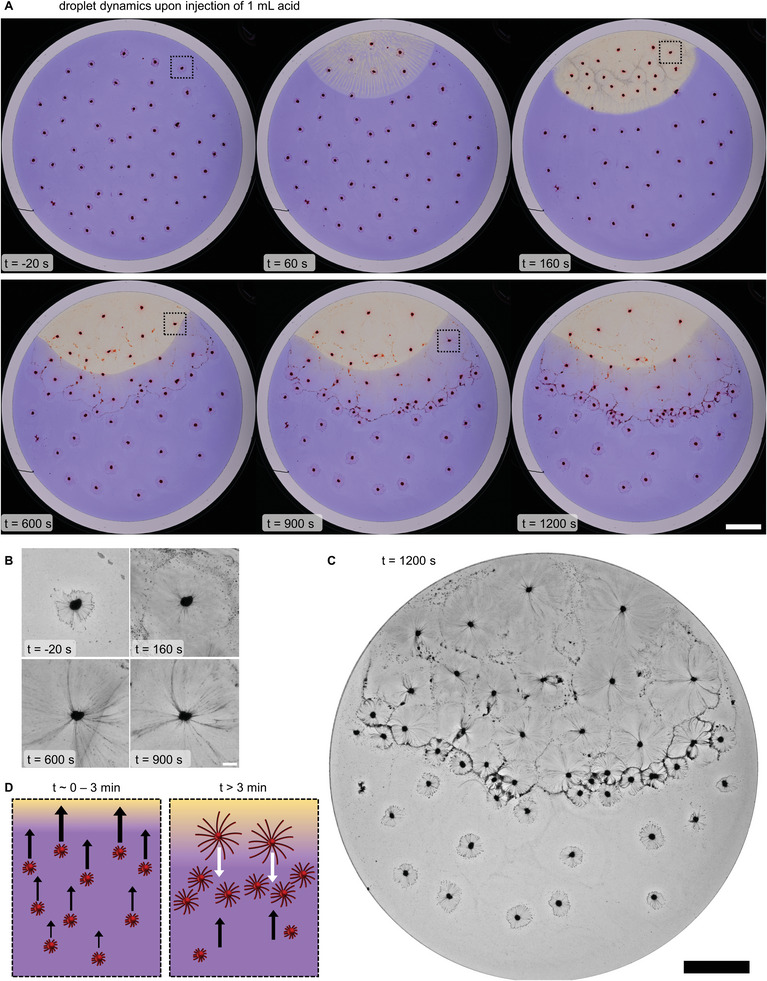
Dynamic self‐organization of the droplets into a “French flag”‐pattern in a pH gradient. A) Photographs of 50 C_12_E_3_ (1 µL) droplets deposited on a solution of 20 mm 1‐octylamine, 7.5 mm DMMA, and 0.0025 wt.% bromocresol purple at pH 7 (100 mm PB, 35 mL). 1 mL HCl (2 m) is injected into the solution at the top region of the dish (t = 0). Initially, droplets close to the acid injection spot accumulate in the acidic, yellow region. Subsequently, droplets in the acidic region start to repel each other. Finally, a semicircular region of high droplet density emerges: a concentrated droplet wave front that separates two regions of low droplet density. The scale bar indicates 20 mm. B) Blue channel, contrast‐enhanced sections corresponding to the dashed regions indicated in (A). The scale bar indicates 2 mm. C) Blue channel, contrast‐enhanced photograph displaying the differences in filament corona size between droplets in the acidic region, the wave front, and the basic region. The scale bar indicates 20 mm. D) Schematic representation of the collective droplet behavior in a pH gradient. Initially, the myelin growth is suppressed due to presence of amide surfactant **1**, and the injected acid (yellow) leads to a global Marangoni flow transporting the droplets towards the acid. Next, local Marangoni flow generated by droplets in the acidic region resumes, as indicated by the increased size of the myelin coronas, and they start to repel neighboring droplets. As a result, most droplets accumulate in a region where the global (black arrows) and local Marangoni flows (white arrows) balance out.

We trigger spatial differentiation of the droplets by creating a pH gradient in the aqueous solution. After deposition of 50 C_12_E_3_ droplets, we slowly inject HCl (2 m, 1 mL) with the dispensing robot at the bottom of the solution to minimize buoyancy convection due to the higher density of the HCl solution. Control experiments in which NaCl (2 m) instead of HCl was injected, demonstrate that buoyancy convection leads to very slow migration of droplets towards the injection spot, but does not affect myelin growth (Figure S6 and Movie S3, Supporting Information). The incorporation of phosphate buffer (100 mm) and the large size of the glass dish ensure that diffusive spreading of the acid is slow. To visualize the resulting pH gradient, we incorporate the pH indicator bromocresol purple in the aqueous solution, which switches color from purple (pH > 6.0) to yellow (pH < 6.0). Immediately after injection of the acid, the pH decreases significantly in an oval‐shaped region as indicated by the solution turning yellow (**Figure** **5A**).

Immediately after its addition, the acid acts as a chemoattractant to the C_12_E_3_ droplets: the droplets in the top area of the dish move rapidly towards the acid and accumulate in the acidic region, as shown in Figure 5A (t = 160 s) and Movie S4 (Supporting Information). Next, the droplets that have accumulated in the acidic region start to grow longer myelins (Figure 5B). Moreover, these droplets become mutually repulsive as indicated by the increase in the droplet–droplet distance inside the acidic region (Figure 5A at t = 600 s). At the same time, droplets that have not reached the acidic region continue to slowly move in that direction. The combination of this persistent (weak) attraction of the droplets by the acid and the mutually repulsive interactions between droplets that arrive in the acidic region results in a striking droplet configuration: ≈20 min after injection of acid, a droplet wave front emerges at the center of the dish that separates a region of droplets with large myelin coronas from a region of droplets with small myelin coronas. This spatially differentiated myelin growth is clearly revealed in blue‐channel, contrast‐enhanced images (Figure 5C). The self‐organized patterns are not equilibrium patterns as can be seen in recordings at t > 20 min (Movie S4, Supporting Information): the pH gradient declines over time and concomitantly, ambient disturbances start to dominate the droplet migration patterns.

The spontaneous organization of the surfactant droplets is a collective effect, i.e., a result of all droplet interactions. Whereas at the start of the experiment, before acid is added, the droplets move rather independently over the interface of the solution, the addition of acid triggers a drastic change in the overall interactions between the droplets. First, the acid hydrolyzes amide surfactant **1**, increasing the surface tension in the acidic region, thereby leading to a global Marangoni flow in the direction of the acidic region (left panel in Figure 5D). Second, droplets that arrive to the acidic, surfactant **1**‐poor region resume the generation of local Marangoni flow as indicated by the start of myelin growth. These local Marangoni flows push away neighboring droplets, so that few droplets remain in the acidic region. Droplets that have not arrived at the acidic region or droplets that are being repelled by their neighboring droplets closer to the spot where acid was injected, also resume generation of local Marangoni flow, but to a lesser extent: their myelin coronas grow less. Lastly, droplets far away from the acidic region are only attracted by the global Marangoni flow: their myelin coronas do not grow over the time course of the experiment (right panel Figure 5D). The self‐organization of droplets does not strongly depend on the amount of droplets present in the dish, as indicated by the same pattern formation in control experiments on 25 or 75 C_12_E_3_ droplets (Figure S7, Supporting Information). Furthermore, we observed that the C_12_E_3_ droplet organization and myelin growth is not affected by pH when DMMA, required to form the strong amide surfactant **1**, is omitted from the system (Figure S8, Supporting Information). Taken together, the droplets translate the positional information established by the global pH gradient by differentiating into three morphologically different regions due to the presence of the external pH gradient.

### 2.5 Effect of Intensity of pH Gradient on Droplet Positioning

**Figure 6:**
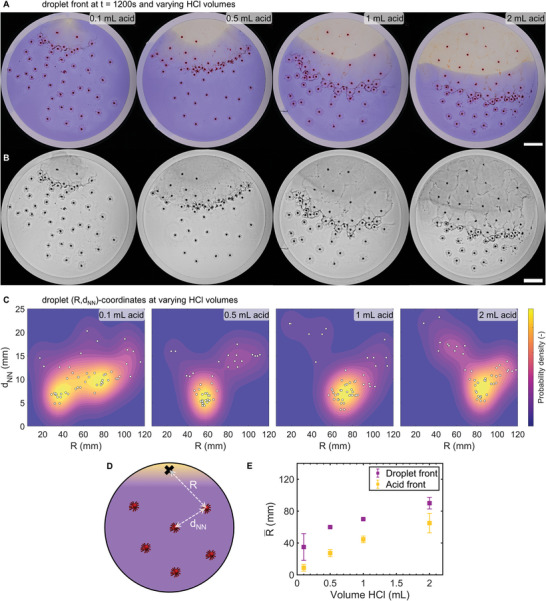
Position of droplet wave front depends on intensity of pH gradient. A) Photographs of 50 C_12_E_3_ droplets (1 µL) deposited on a solution of 20 mm 1‐octylamine, 7.5 mm DMMA, and 0.0025 wt.% bromocresol purple at pH 7 (100 mm PB, 35 mL), 20 min after varying amounts of 2 m HCl have been injected into the solution; 0.1 mL (pH 6.8 after mixing); 0.5 mL (pH 6.3); 1.0 mL (pH 5.6) and 2.0 mL (pH 2.0). The scale bar indicates 20 mm. B) Blue channel, contrast‐enhanced photographs displaying the differences in myelin corona size between droplets in the acidic region, the wave front, and the basic region. The scale bar indicates 20 mm. C) 2D probability density of droplet coordinates (R,d_NN_, see D) at t = 1200 s corresponding to experiments in A,B). White data points indicate the (R,d_NN_) coordinates of the 50 C_12_E_3_ droplets. D) Schematic representation of (R,d_NN_) coordinates. R represents the distance of droplet to acid injection spot, d_NN_ the nearest neighbor distance. E) Average position $\bar{R}$ of droplet wave front (purple) and acid front (yellow). Purple $\bar{R}$ values correspond to the R‐coordinate of the peaks in the 2D probability density in D). Error bars indicate standard deviation (*n* = 3 separate experiments).

To study how the intensity of the pH gradient affects the positioning of the droplet wave front, we varied the amount of acid added to the solution (0.1, 0.5, 1, 2 mL HCl, 2 m), resulting in final pH values, upon full mixing, of 6.8, 6.3, 5.6 and 2.0, respectively. In all experiments, we deposit 50 C_12_E_3_ droplets at the surface of the aqueous solution (20 mm 1‐octylamine, 7.5 mm DMMA, 0.0025 wt.% bromocresol purple, 100 mm PB pH 7). Next, we slowly inject acid (t = 0 s), at the same position in the solution and over the same injection time (30 s) for all experiments. As anticipated, both the size of the yellow, acidic region and the number of droplets accumulating in the acidic region increases with increasing amount of acid injected into the solution (**Figure** **6A**; Movies S5 and S6, Supporting Information). Similarly, the number of droplets that are not significantly affected by the injection of acid, i.e., droplets that keep a constant myelin corona over the duration of the experiment, decreases when more acid is injected (Figure 6B). Importantly, we find that in all experiments, a droplet wave front emerges after ≈20 min, of which the position shifts away from the acid injection spot upon increasing the volume of injected acid.

To quantify how much the droplet wave front shifts upon increasing the amount of acid injected into the solution, we reasoned that the droplets comprising the wave front share two quantitative properties: 1) they are close to neighboring droplets, and 2) they are all positioned approximately at an equal distance away from the spot where acid was injected. To determine the position of the center of the wave front, we first track the (x,y)‐position of each droplet with a particle tracking algorithm.^[^ ^32^ ^]^ Next, we calculate the distance between each droplet and the position where the acid was injected (R), and we approximate the local droplet density at each droplet position by calculating the nearest neighbor distance for each droplet (d_NN_), (Figure 6D). Indeed, when we plot the (R,d_NN_)‐coordinates for each droplet, the data clusters at low d_NN_ (≈7.5 mm) and at R values increasing with increasing amount of injected acid (Figure 6C). Finally, we extract the position of the droplet wave front center $\bar{R}$ by finding the maximum in the (R,d_NN_) probability density estimate based on all 50 droplet coordinates. When the probability density estimate has more than one maximum, as is the case in the left‐most panel in Figure 6C, we select the peak at the lowest d_NN_ value. As shown in Figure 6E, $\bar{R}$ increases with the volume of HCl solution added, similar to the position of the acid front.

## 3 Conclusion

We demonstrate the spatial organization of floating C_12_E_3_ surfactant droplets into “French flag”‐patterns based on an external pH gradient. The droplets generate local, mutually repulsive Marangoni flows, visualized by myelin filaments that grow from the droplet periphery, depending on the presence of an amide surfactant that is hydrolyzed under acidic conditions. This competitive amide surfactant adsorbs stronger to the air‐water interface than C_12_E_3_ and thereby inhibits the local Marangoni flows. When exposed to an external pH gradient, the droplets experience two consecutive effects: 1) a global Marangoni flow arises from the basic side towards the acidic side of the pH gradient, attracting C_12_E_3_ droplets towards the acidic side; 2) consecutively, the local Marangoni flows are reestablished by the acid‐exposed C_12_E_3_ droplets and lead to mutual repulsion between neighboring droplets. This combination of attractive global Marangoni flow and repulsive, position‐dependent local Marangoni flows leads to spatial differentiation of the droplet population into three regions of low–high–low droplet density, showing a unique strategy to establish “French flag”‐patterns based on droplets and surfactants. Furthermore, the morphological difference between the two low droplet density regions is clearly revealed by the difference in myelin growth in either region: long myelins in the acidic region, short myelins in the basic region.

Marangoni flow offers an attractive design principle to translate chemical concentration gradients into motion, due to the direct coupling between surfactant concentration gradients and liquid flow. Moreover, the fast equilibration of surface tension to concentration allows for rapid adaptation to changing concentration gradients, as we observed with a field of C_12_E_3_ droplets that consecutively form a wave front, spread and reform a wave front upon addition of acid, base and acid, respectively (Figure S9, Supporting Information). Importantly, applicability of Marangoni flow is not limited to 2D interfaces, as immersed droplets can be engineered to self‐organize based on internal Marangoni flows.^[^ ^33^, ^34^, ^35^ ^]^ Alternative mechanisms in which the driving force responsible for the transport and the transported cargo are susceptible to externally imposed chemical gradients, such as buoyancy convection^[^ ^36^ ^]^ or diffusiophoresis^[^ ^37^ ^]^ could lead to pattern formations analogous to what we have presented in this work. Droplets are especially attractive as active transport cargos in such a scenario, due to the easy control over their chemical composition and physico‐chemical responsiveness. Ultimately, we envision that the concept of competing local and global (Marangoni) flows, combined with droplet swarms that control or self‐generate concentration gradients, allows for adaptation of functional droplet‐based materials in response to their environment.

## 4 Experimental Section

### Materials

Tri(ethylene glycol) monododecyl ether (C_12_E_3_, > 95%) and 2,3‐dimethylmaleic anhydride (DMMA, > 98%) were purchased from TCI Chemicals. Hydrochloric acid (2 m in water), sodium dihydrogen phosphate (99%) and sodium chloride (> 99%) were purchased from Fisher Scientific. Deuterium oxide (99.9%), disodium hydrogen phosphate (> 99%), 1‐octylamine (99%), and Oil Red O were purchased from Sigma–Aldrich. 3‐(Trimethylsilyl)propanoic‐2,2,3,3‐d_4_ acid (TSP‐d_4_, > 97.5%) was purchased from Thermo Scientific. 2‐propanol was purchased from VWR. All materials were used as received.

### Methods

*Surface Tension Measurements*: Surface tension measurements were performed on a force tensiometer (Biolin Scientific Sigma 701) with a platinum Du Noüy ring (wetting length = 120.4 mm). The ring was cleaned between measurements by rinsing with ethanol and heating with a flame torch until glowing red hot. For each measurement, a Petri dish (Falcon, 35 mm) was filled with 6.0 or 7.0 mL solution. All solutions containing 1‐octylamine and/or DMMA were thoroughly vortex‐mixed for ≈30 s and subsequently sonicated for ≥ 20 min until fully dissolved.

*Microscopy Experiments on Myelin Corona Growth*: Myelin growth of single C_12_E_3_ droplets was recorded with an inverted optical microscope (Olympus IX73) equipped with a 1.25x magnification objective (Olympus Plan Apo N 1.25×0.04 NA). Images were captured in dark field mode at 1 fps with a CMOS camera (Point Grey Grasshopper3). For each measurement, the lid of a Petri dish (Falcon, 35 mm) was filled with 5.5 mL solution. Next, a C_12_E_3_ droplet (1 µL) was manually pipetted slightly under the surface of the solution to prevent the droplet from bursting. Acid (2 m HCl) is also pipetted under the surface of the solution to limit disturbance of the myelin growth.

*Quantitative ^1^H‐NMR measurements to Determine Concentration Amide Surfactant* ***1***: The time‐dependent concentration of amide surfactant **1** was measured with ^1^H‐NMR, by calculating the ratio between the peak integrals of the (C_7_H_15_‐C**H_2_**‐NH‐R)‐protons and that of the (C_7_H_15_‐C**H_2_**‐NH_3_^+^)‐protons of 1‐octylamine. We obtain the concentration of amide surfactant **1**, by assuming that the total concentration 1‐octylamine and amide surfactant **1** is 20 mM. The samples were prepared from a 90:10 mixture of 1‐octylamine (22.2 mm), DMMA (5.5 mm) in sodium phosphate buffer (100 mm, pH 7) in H_2_O, and 3‐(trimethylsilyl)propanoic‐2,2,3,3‐d_4_ acid (10 mm; TSP‐d_4_) in D_2_O. TSP‐d_4_ was included to indicate 0.00 ppm. A small amount of this solution was used to determine the amount of HCl required to set the pH to 6.5. Next, ^1^H‐NMR measurements were performed for both samples at pH 7.0 and 6.5, using a water peak suppression program (Bruker Avance III, 500 MHz).

*Droplet Dispensing Robot*: The droplet dispensing robot (developed by Labm8) comprises 7 motorized axes that are controlled by a controlling unit (Labm8, M8001.1). C_12_E_3_ and HCl solution were dispensed from 2 blunt, stainless‐steel needles (Fisnar, inner diameter: 0.8 mm; 21 Ga). The position of the dispensing needles was independently actuated by 2 motorized axes in z, 1 motorized axis in x, and 2 parallel motorized axes in y (Figure 4A,B). The C_12_E_3_ dispensing needle was connected to a glass syringe (Hamilton, 1.5 mL) and the HCl solution dispensing needle to a glass syringe (Hamilton, 25 mL) with PTFE tubing. The syringes were actuated by motorized axes, i.e., syringe pumps (Labm8, M8003.1). All motors were calibrated stepper motors, so that the imposed actuation in steps can be converted to movement in millimeters or volume in microliters. The motorized axes were mounted onto an aluminum base plate. The entire setup was mounted on an anti‐vibration optical table and levelled using adjustable legs.

To enhance the visual contrast between C_12_E_3_ droplets and the aqueous solution in recorded images, the dye Oil Red O (20 mg mL^−1^) is incorporated into the surfactant solution. To deposit C_12_E_3_ droplets precisely at the air–water interface, a 2 wire analog electrical probing circuit connected to the stainless‐steel needle was used that dispensed the droplets and a copper wire positioned inside the aqueous solution. When the needle and the surface of the aqueous solution come into contact, a signal is registered (above a set threshold), which is interpreted by the robot to stop motion in the z‐direction after which it starts dispensing C_12_E_3_. In all the experiments, solutions containing sodium phosphate buffer (100 mm) were used, which was sufficient for the probe to detect an over‐threshold event. Droplets were deposited on a solution contained in a thin, custom‐made glass dish (inner diameter: 131 mm, thickness: 2 mm) held in place over a circular hole in the base plate.

The sample was illuminated with a LED panel (Viltrox VL‐200T, color temperature 5600 K) and was diffused with a thin (3 mm) sheet of frosted polymethyl methacrylate. Images were captured with a mirrorless camera (Nikon Z5) and macro lens (Laowa 100 mm f/2.8). For all experiments the camera settings were as follows, ISO: 100, shutter speed: 1/25 s, aperture: f22, white balance: daylight, capture rate: 1 frame every 20 seconds.

*Deposition Droplet Field*: C_12_E_3_ droplets were deposited in the sunflower seed arrangement to ensure homogeneous spreading in the circular dish. The position of droplet *i* in polar coordinates (*r_i_*,θ_*i*_) with (0,0) was computed at the center of the dish as:

(1) $$r_{i}=r_{\max}\;\times \sqrt{i-1/2}\;/\sqrt{\left(n-b+1\right)/2}\;$$

(2) $$\theta_{i}=\;2\pi \;i/\left(\phi -1\right)$$

with *r_max_* = 56.2 mm the radius of the deposition zone, *n* the total number of droplets, *b* = 7 the number of droplets on the boundary of the deposition zone, and ϕ the golden ratio $(1+\sqrt{5})/2$. *r_max_* was chosen so that droplets are not deposited closer to the edge of the dish than 0.5 times the average droplet‐droplet distance, i.e., $r_{dish}-\;r_{max}=\;\sqrt{A_{dish}/\left(n\times \pi \right)}$, where *A_dish_*/*n* is the average area per droplet. The droplet coordinates were computed, converted to robot coordinates, and exported to a G‐code file. The G‐code file also includes the dispensing flow rate and needle movement rate (x,y: 500 mm min^−1^, z: 100 mm min^−1^). The dispensing flow duration for C_12_E_3_ was set to 2 s (0.5 µL s^−1^), the dispensing flow duration of the HCl solution was set to 30 s for all volumes of dispensed HCl solution.

*Generation of pH Gradient*: To generate a pH gradient, a HCl solution (2 m) was injected with the droplet dispensing robot into the aqueous solution at a fixed position in the dish, ≈1 mm offset from the edge of the dish. To minimize buoyancy convection due to the density difference of the HCl solution (ρ = 1.04 ± 0.00 g mL^−1^) and that of the aqueous solution [ρ = 1.01 ± 0.01 g mL^−1^, DMMA (7.5 mm), 1‐octylamine (20 mm), sodium phosphate buffer pH 7 (100 mm), bromocresol purple (0.0025 wt.%)], the HCl solution was injected at the bottom of the solution, ≈0.5 mm above the bottom of the dish. Both solution densities were measured by weighing known volumes of these solutions (*n* = 10).

*Determination of Droplet Positions*: To determine the droplet positions based on the recorded time‐lapse frames, the MATLAB implementation of a particle tracking algorithm originally developed by Crocker and Grier was used.^[^ ^32^, ^38^ ^]^ Here, a comprehensive overview of the analysis steps is given with algorithm and MATLAB function names in italics.

Step 1 – *Pre‐processing*: First, parts of the original frames outside the dish were removed, including the dish edges, by binarizing the frames (*imbinarize*) and cropping the regions of intensity 0 (*imcrop*). Second, the red and green channels were discarded from the RGB frames as the red C_12_E_3_ droplets contrast most against the background in the blue channel. Next, the color scale of the resulting frame (*imcomplement*) was inverted so that the droplets appear bright against a dark background. Lastly, the image to 1/9th the original size (*imresize*) was resized to speed up computation.
Step 2 – *Droplet tracking*: The pre‐processed frames were sequentially filtered using a bandpass filter (*bpass*) with high frequency cutoff set to 11 pixels and low frequency cutoff set to 21 pixels. Next, droplet positions in each frame were identified (*findfeatures*) based on the size (35 pixels) and minimum pixel brightness (5) of bright spots in the filtered frames. Finally, the identified spots were evaluated and another filter was applied (spot brightness > 3500, spot size < 120 pixels, spot eccentricity < 0.15.
Step 3 – *Computation of droplet coordinates* *R* *and* *d_NN_* *and probability density estimation*: Droplet coordinates in x,y were converted to coordinates *R*  = ((*x_i_* − *x_acid_*)^2^  + (*y_i_* − *y_acid_*)^2^)^1/2^, i.e., the distance between droplet *i* and the acid injection spot, and the nearest neighbor distance  *d_NN_* = min [((*x_i_* − *x_j_*)^2^ + (*y_i_* − *y_j_*)^2^) ^1/2^]. Subsequently, the droplet probability density was estimated using a 2D kernel estimator (*mvksdensity*) with bandwidth 10 and 3 in *R* and *d_NN_*, respectively. Finally, the probability densities were plotted in a filled contour plot (*contourf*).

*Computation Radius of Filament Coronas*: To determine the sizes of the filament coronas based on the recorded time‐lapse frames, image analysis was performed with MATLAB. First, the custom algorithm used for analyzing optical microscopy images and visualizing the filament corona growth (Figure 3C) is described. Second, the algorithm used for analyzing camera images and visualizing the filament corona variation as a function of the DMMA concentration (Figure 4F) is described.

1. *Microscopy image analysis*: First, the C_12_E_3_ droplet was identified by binarizing the microscopy frames (*imbinarize*), inverting the color scale (*imcomplement*), eroding (*imerode*) and dilating the image (*imdilate*) and finally filtering the largest object (*findfeatures*). The same sequence of steps was applied to find the filament corona, with as only difference the binarization threshold (0.5 for the droplet, 0.55 for the filament corona). Next, the droplet and filament corona were combined and bounding box size was computed (*regionprops*). The filament corona radius, including droplet, was calculated as 0.5x the average between height and width of the bounding box dimensions. This algorithm was applied to every 10^th^ recorded frame, i.e., once every 10 s.
2. *Camera image analysis*: First, the dish edges were removed from the original frames by binarizing the frames (*imbinarize*) and cropping the regions of intensity 0 (*imcrop*). Next, the droplet positions were found by applying the droplet tracking algorithm described above. The corona radius was computed by subsequently enhancing the contrast of the cropped blue channel image (*adapthisteq*), binarizing (*imbinarize*), inverting the color scale (*imcomplement*), identifying the objects that belong to the droplet position coordinates (*bwselect*), and calculating the bounding box of the resulting objects (*regionprops*).

## Conflict of Interest

Max Derks is the owner of Labm8 (www.labm8.io/), and has contributed to the development of the droplet deposition robot used in this work, which is partly composed of commercially available Labm8 equipment. The other authors have no interest in Labm8, and also no other conflict of interest to report.

## Supporting information

Supporting Information

Supplemental Movie 1

Supplemental Movie 2

Supplemental Movie 3

Supplemental Movie 4

Supplemental Movie 5

Supplemental Movie 6

## Data Availability

The data that supports the findings of this study are available in the supplementary material of this article.
