# Supplementary material for: Positional Information‐Based Organization of Surfactant Droplet Swarms Emerging from Competition Between Local and Global Marangoni Effects
Source: Small. 2024 Aug 21;20(47):2403720. doi: 10.1002/smll.202403720 (PMC11579963; doi:10.1002/smll.202403720)
Supplement: Supplementary file 1 — Supporting Information [file SMLL-20-2403720-s001.docx]

Supporting Information

Positional Information-based Organization of Surfactant Droplet Swarms Emerging from Competition between Local and Global Marangoni Effects.

Pieter J. de Visser, Mink Neeleman, Pim F. J. Dankloff, Max T. G. M. Derks & Peter A. Korevaar*

**Table of Contents**

Supporting Figures

Figure S1: pH-dependent surface tension measurements of DMMA

Figure S2: ^1^H-NMR spectrum of **1**

Figure S3: C_12_E_3_ myelin growth on DMMA solution

Figure S4: Time-dependent surface tension measurements of 5 mm amide surfactant **1**

Figure S5: Corona sizes at varying DMMA concentrations

Figure S6: NaCl control experiment droplet swarm

Figure S7: Self-organization of 25 and 75 C_12_E_3_ droplets in pH gradient

Figure S8: Self-organization of 50 C_12_E_3_ droplets at pH 4 and 7

Figure S9: Reversible self-organization of C_12_E_3_ droplets upon acid-base-acid addition

Description of Supporting Movies S1 – S6


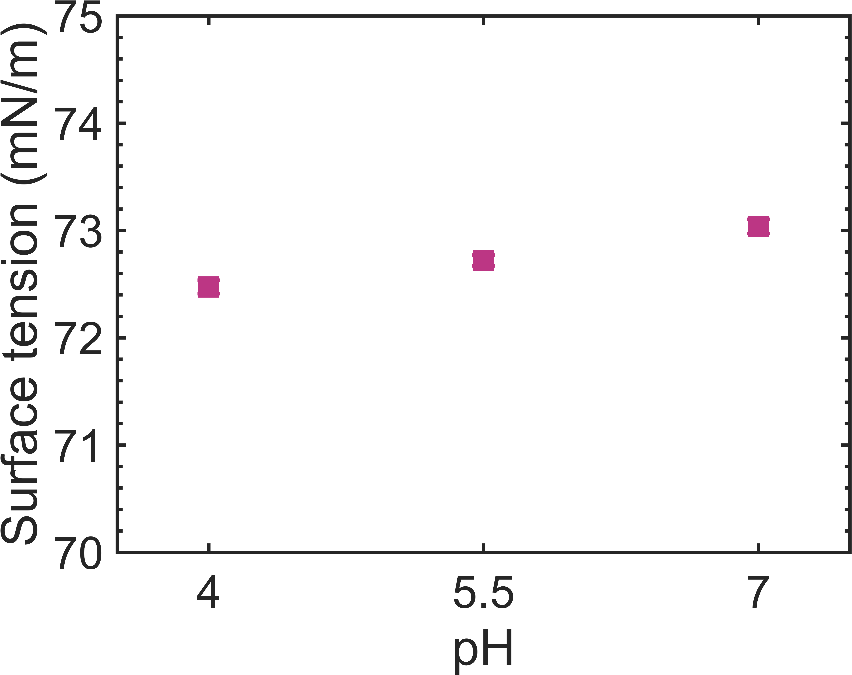


**Figure S1.** pH-dependent surface tension measurements of 5 mm DMMA in water (*n* = 2). The error bars are smaller than data indicators due to the low standard deviation between measurement data.


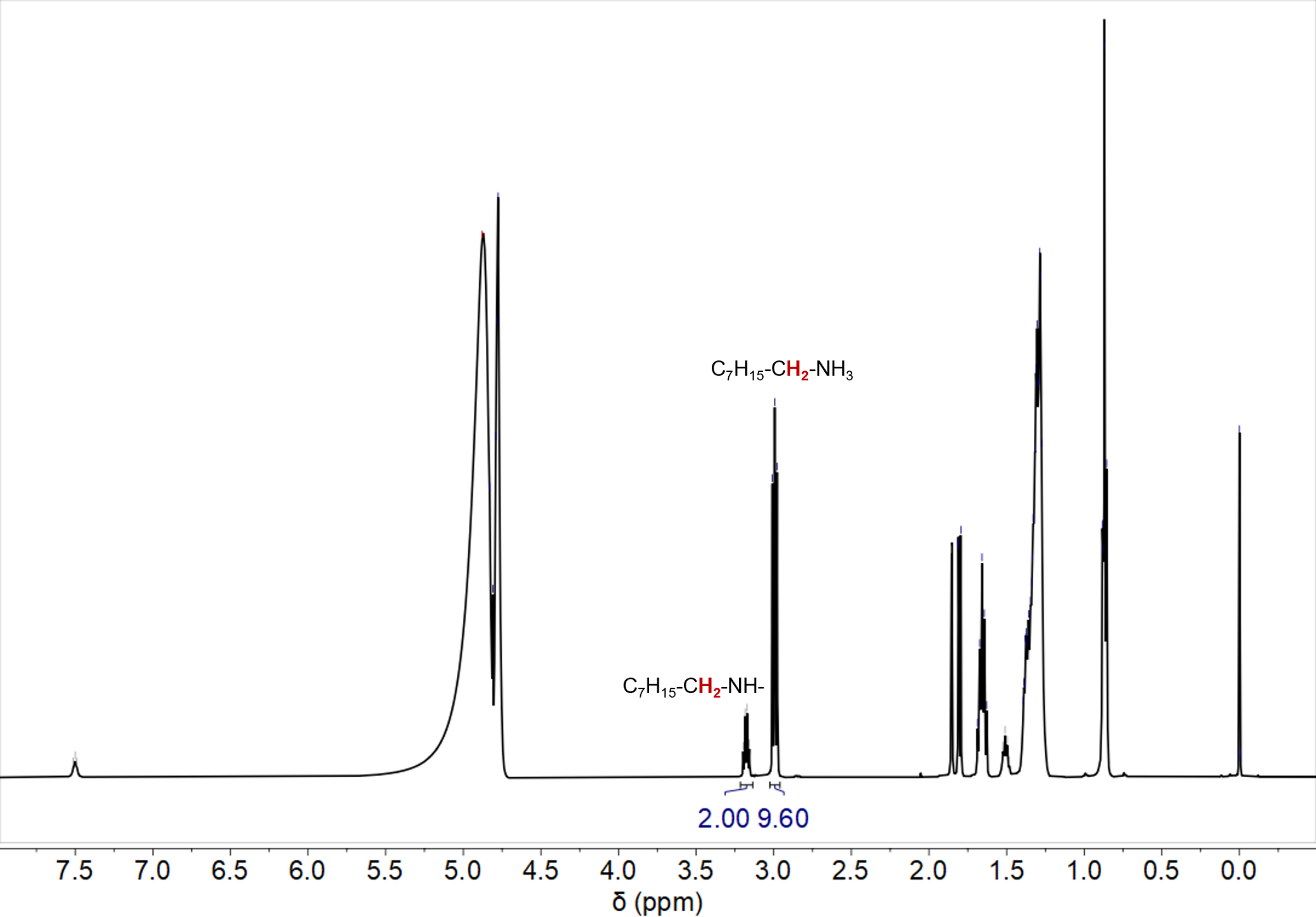


**Figure S2.** ^1^H-NMR spectrum of 5 mm DMMA, 20 mm 1-octylamine in 100 mm PB pH 7 in a 90:10 H_2_O:D_2_O solution, with TSP-d_4_ (1 mm). The protons neighboring the amine group of 1-octylamine at 3.0 ppm and the corresponding protons of amide surfactant **1** at 3.2 ppm are indicated in red.


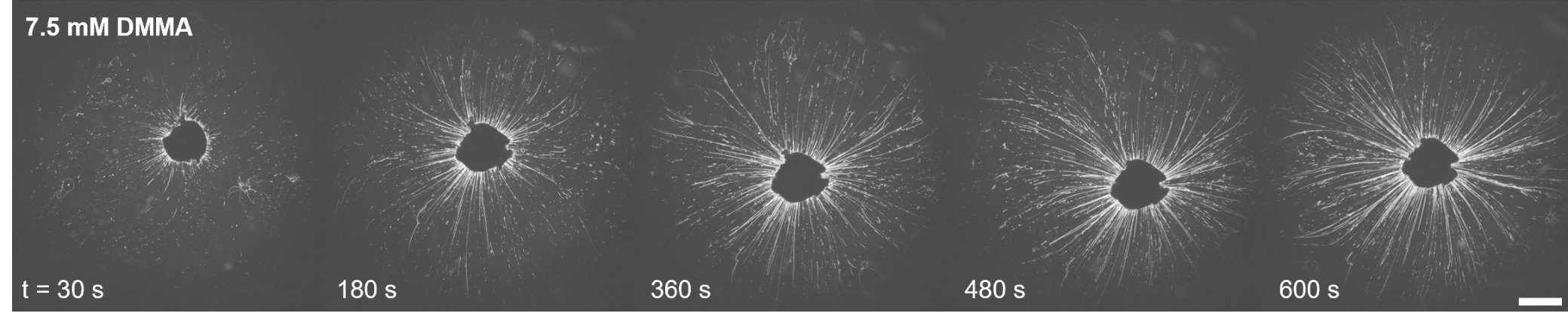


**Figure S3.** Optical microscopy recordings of 1 µL C_12_E_3_ deposited on a solution with 7.5 mm DMMA at pH 7 (100 mm PB). Myelin growth is slightly hampered by DMMA, although the growth does not halt as compared when 1-octylamine is incorporated in the solution (cf. Figure 2c). The scale bar indicates 2 mm.


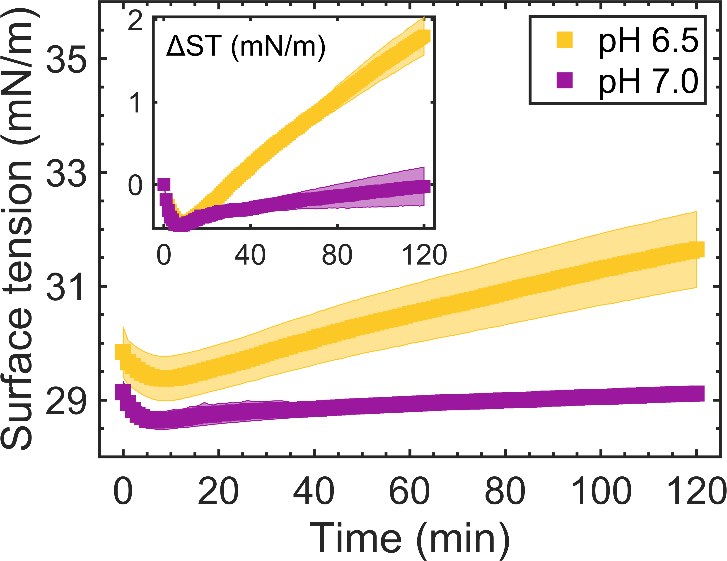


**Figure S4.** Time-dependent surface tension, measured on solutions of 20 mm 1-octylamine and 5 mm DMMA at pH 6.5 and 7.0 (100 mm PB). The slow increase in surface tension at pH 6.5 indicates that amide surfactant **2** slowly hydrolyses. The inset shows ΔST = ST(t) – ST(0). Error bars indicate standard deviation (*n =* 2).


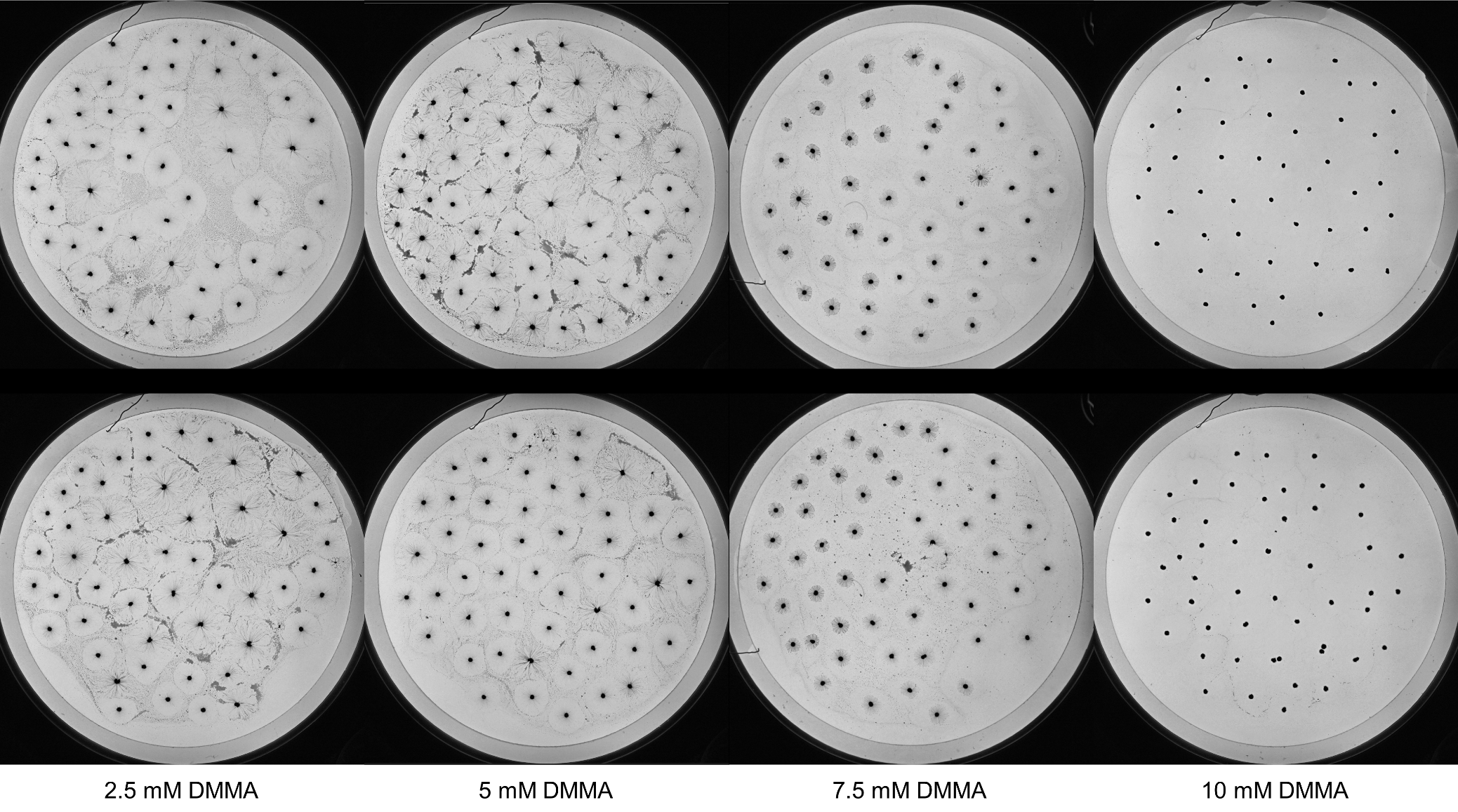


**Figure S5.** Photographs of 50 1 μL C_12_E_3_ droplets 5 min after deposition on top of solutions with varying concentrations DMMA and 20 mm 1-octylamine, 0.0025 wt% bromocresol purple at pH 7 (100 mm PB). To enhance visibility of the filaments, the blue channel of the original RGB images was contrast enhanced. The scale bar indicates 10 mm.


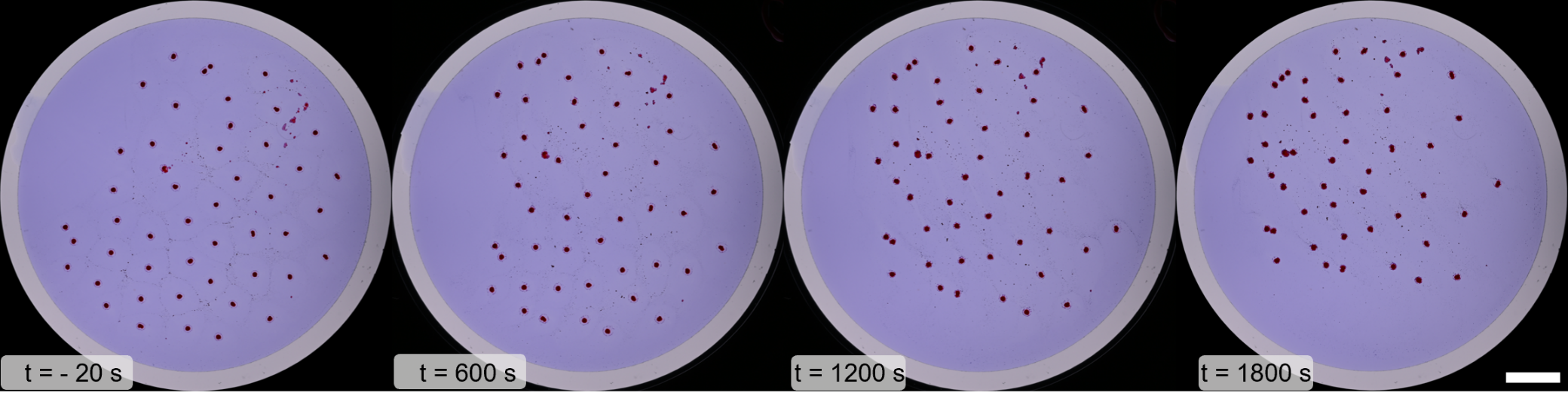
**Figure S6.** Photographs of 50 C_12_E_3_ droplets (1 μL) deposited on a solution of 20 mm 1-octylamine, 7.5 mm DMMA, and 0.0025 wt% bromocresol purple at pH 7 (100 mm PB, 35 mL). At t = 0 s, 1 mL NaCl (2 m) was injected with the droplet dispensing robot at the top of the dish. No myelin growth is observed. There is a small drift of the droplets towards the spot where salt was injected. The scale bar indicates 20 mm.


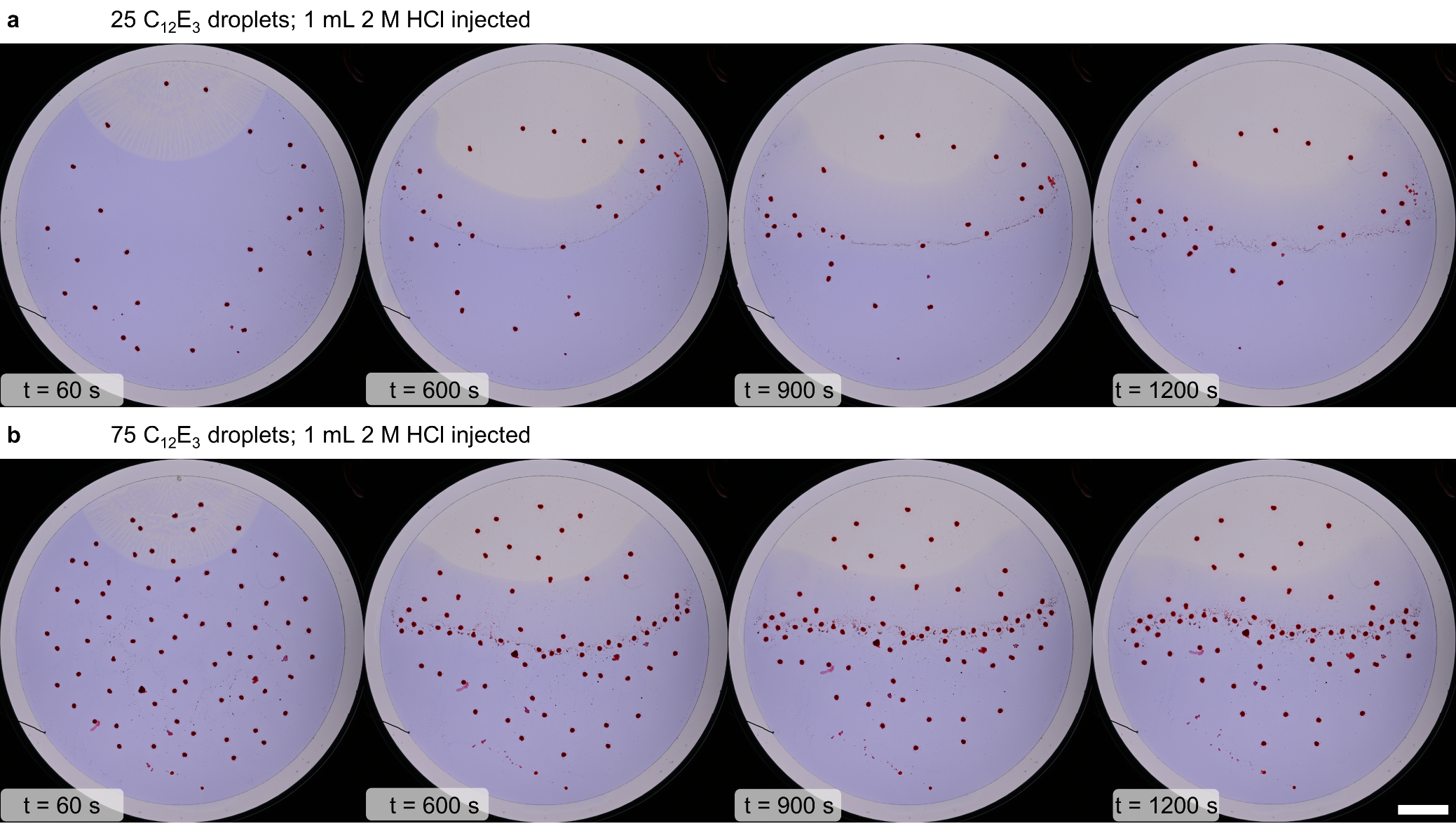


**Figure S7.** Photographs of 25 (in a) and 75 (in b) C_12_E_3_ droplets (1 μL) deposited on a solution of 20 mm 1-octylamine, 7.5 mm DMMA, and 0.0025 wt% bromocresol purple at pH 7 (100 mm PB, 35 mL). The scale bar indicates 20 mm.


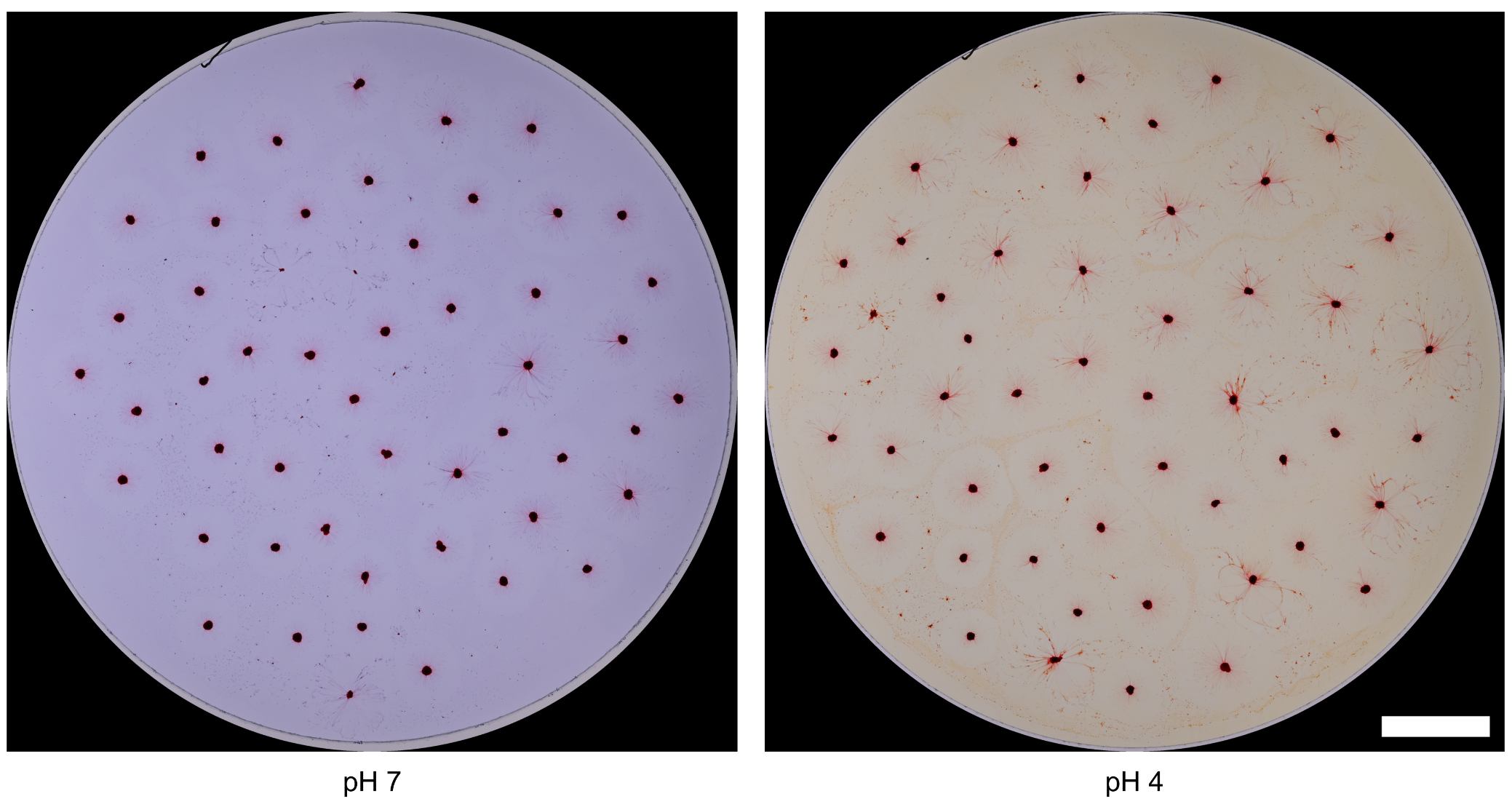


**Figure S8.** Photographs of 50 C_12_E_3_ droplets (1 μL) deposited on a solution of 20 mm 1-octylamine, 0.0025 wt% bromocresol purple, and 100 mm PB at pH 7 and pH 4. The scale bar indicates 20 mm.


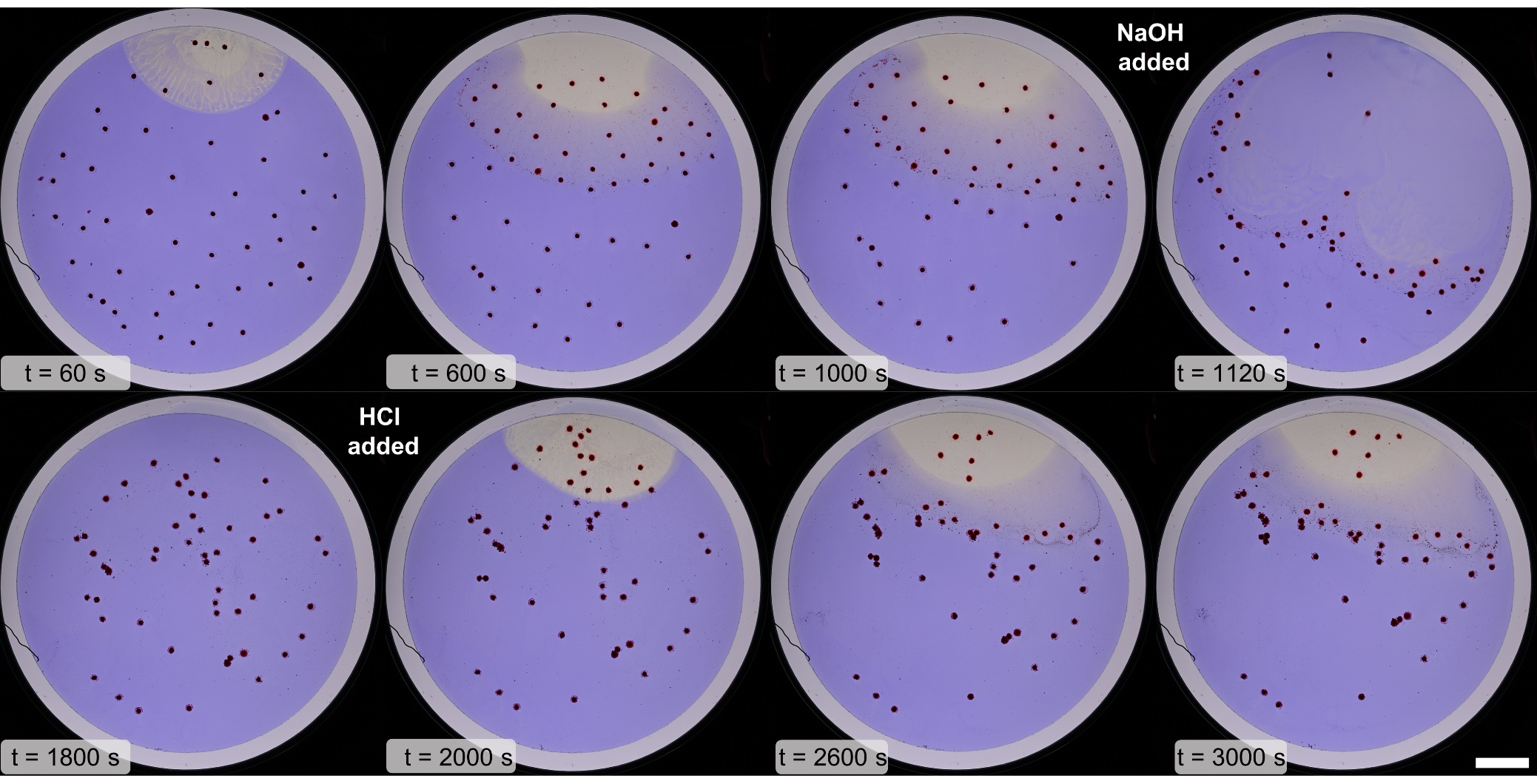


**Figure S9.** Photographs of 50 C_12_E_3_ droplets (1 μL) deposited on a solution of 20 mm 1-octylamine, 7.5 mm DMMA, 0.0025 wt% bromocresol purple, and 100 mm PB at pH 7. At t = 0 and t = 1880 s, 0.5 mL 2 m HCl was added to the dish. At t = 1080 s, 0.5 mL 2 m NaOH was added to the dish. The scale bar indicates 20 mm.

**Description of Supporting Movies S1 – S6**

NB. Time is indicated in (HH:)MM:SS format in all Supporting Movies.

*File Name: Movie S1 – Optical microscopy recording corresponding to Figures 2c-d and S3*

C_12_E_3_ myelin growth on aqueous solutions with amide surfactant **2** (left panel), with precursor 1-octylamine (middle panel), or with precursor DMMA (right panel). Myelin growth only fully halts if amide surfactant **1** is present. DMMA destabilizes the myelins, but myelins continue to grow. All solutions are set to pH 7 (100 mm PB).

*File Name: Movie S2 – Optical microscopy recording corresponding to Figure 3a*

Reactivation of C_12_E_3_ myelin growth on an aqueous solution with amide surfactant **1** (7.5 mm DMMA, 20 mm 1-octylamine) upon addition of HCl solution at t = 250 s. Initially, the pH of the solution is set to pH 7 (100 mm PB), and after addition of HCl, the pH is measured to be 6.5.

*File Name: Movie S3 – Time lapse recording corresponding to Figure S4*

Control experiment for droplet swarm self-organization. NaCl (2 m, 1 mL) was injected into the solution instead of HCl at t = 0 s. Over the course of approx. 30 minutes myelins do not grow, but the droplets slowly drift towards the spot where the NaCl solution was injected, and some droplets clump together.

*File Name: Movie S4 – Time lapse recordings corresponding to Figure 5a*

Full color (RGB, left) and contrast enhanced, blue channel (right) recordings of the self-organization of 50 C_12_E_3_ droplets in a pH gradient. The aqueous solution contains 7.5 mm DMMA, 20 mm 1-octylamine, 0.0025 wt% bromocresol purple and is set to pH 7 (100 mm PB). At t = 0 s, 1 mL HCl (2 m) is injected into the solution with the droplet dispensing robot. The ‘French flag’-pattern is established approx. 20 minutes after addition of the acid.

*File Name: Movie S5 – Time lapse recordings corresponding to Figures 6a-b*

Full color (RGB, top row) and contrast enhanced, blue channel (bottom row) recordings of the self-organization of 50 C_12_E_3_ droplets in a pH gradient. The aqueous solution contains 7.5 mm DMMA, 20 mm 1-octylamine, 0.0025 wt% bromocresol purple and is set to pH 7 (100 mm PB). At t = 0, the indicated amount of HCl (2 m) is injected into the solution with the droplet dispensing robot.

*File Name: Movie S6 – Time lapse recordings corresponding to Figure 6e*

Full color (RGB) recordings of the self-organization of 50 C_12_E_3_ droplets in pH gradients of different intensities (0.1, 0.5, 1, 2 mL HCl solution added). The aqueous solution contains 7.5 mm DMMA, 20 mm 1-octylamine, 0.0025 wt% bromocresol purple and is set to pH 7 (100 mm PB). At t = 0, the indicated amount of HCl (2 m) is injected into the solution with the droplet dispensing robot.
